# Ten-Year Follow-Up of Visual Outcomes After Vitrectomy for Clinically Non-Tractional Diabetic Macular Edema

**DOI:** 10.7759/cureus.95537

**Published:** 2025-10-27

**Authors:** Kazuyuki Kumagai, Eiji Horie, Marie Fukami, Mariko Furukawa

**Affiliations:** 1 Ophthalmology, Kami-iida Daiichi General Hospital, Nagoya, JPN; 2 Ophthalmology, Yata Eye Clinic, Nagoya, JPN

**Keywords:** anti-vascular endothelial growth factor, diabetic macular edema, long-term visual outcomes, phacovitrectomy, vitrectomy

## Abstract

This study evaluates the 10-year visual outcomes of vitrectomy for clinically non-tractional diabetic macular edema (DME). The medical records of 496 eyes of 332 consecutive patients who underwent vitrectomy for DME by one surgeon (NO) between August 1990 and June 2000 were retrospectively reviewed. A standard 20-gauge three-port pars plana vitrectomy was performed. The surgery consisted of phacovitrectomy for phakic eyes with the removal of the posterior hyaloid. The internal limiting membrane was peeled in 181 of the 496 eyes (36.5%). The eyes were divided into four groups based on the duration of the follow-up period (F): F<1 year (n=10), 1 year ≦F<5 years (n=128), 5 years ≦F<10 years (n=201), and 10 years ≦ F (n=157). The decimal visual acuities were converted to the logarithm of the minimum angle of resolution (logMAR) units and also to the corresponding Early Treatment Diabetic Retinopathy Study (ETDRS) letter scores. The main outcomes were the visual acuities. In addition, long-term visual outcomes after anti-vascular endothelial growth factor (anti-VEGF) treatments were investigated from previous reports. The postoperative follow-up period ranged from 4 to 280 months with a mean of 90.9 months. Three groups other than the F<1 year group had similar preoperative characteristics and visual outcomes. The mean letter score of the 10 years ≦F group was 51.9 before surgery, 61.7 at six months, 63.7 at 1 year, 63.4 at two years, 63.0 at three years, 62.6 at five years, and 60.0 at 10 years. The 10-year visual outcomes after vitrectomy for clinically non-tractional DME appeared to be good and stable. Further studies are expected to compare the long-term outcomes of vitrectomy and anti-VEGF treatments. Prospective blinded studies are needed to show efficacy.

## Introduction

Diabetic macular edema (DME) is a major cause of reduced vision in diabetic individuals. Diabetes mellitus is a growing global epidemic [1], and the need for DME treatment is increasing.

The current first-line treatment for DME is intravitreal anti-vascular endothelial growth factor (anti-VEGF) injections [2-6]. However, the use of anti-VEGF agents has some difficulties, especially the need for multiple injections, which will impose both psychological and economic burdens on the individual [7-11]. In addition, there are several reports [12-14] on long-term results, but none on cases followed for more than 10 years. Patients receiving anti-VEGF treatment generally undergo fewer postoperative monitoring examinations and fewer intravitreal injections. Importantly, they have poorer vision than patients in clinical trials [15]. Another difficulty of anti-VEGF treatment is whether the outcomes obtained in other countries will be applicable to patients in Japan, where the racial and genetic demographics are different. All the drugs nowadays undergo multicenter trials. However, such concerns still exist. In addition, the insurance system and access to medical care are also different.

Photocoagulation and vitrectomy were used to treat DME until the early 2000s. A significant relationship between the development of DME and the vitreous has been reported [16-19]. Based on these reports, the use of vitrectomy began in the 1990s. Since then, many reports have been published on the effects of vitrectomy on DME [20-25], and the current consensus is that it is effective for eyes with macular traction. However, its effectiveness on eyes without macular traction has not been definitively determined [26,27].

There has recently been a re-evaluation of the visual outcome after vitrectomy for the treatment of DME [6,28-39], especially for naive cases [40-42]. Comparisons of the visual outcomes of anti-VEGF treatment to those of vitrectomy have been reported in three studies [43-44]. In Japan, Terasaki et al. proposed that conventional laser photocoagulation, vitrectomy, and steroid therapy are also expected to play a role in the treatment of DME [3].

Thus, the purpose of this study was to determine the long-term effects of vitrectomy on eyes with DME without traction. This report extends the follow-up period of the previous report [25] and evaluates the visual outcomes of a 10-year follow-up period. In addition, we tried to evaluate visual outcomes by referring to the outcomes of anti-VEGF treatments from previous reports.

## Materials and methods

This was a retrospective, consecutive case series conducted with the approval of the Ethics Committee of the Kami-iida Daiichi General Hospital. The procedures used conformed to the tenets of the Declaration of Helsinki. All patients signed an informed consent form for the surgery, data collection, and the use of their data for research studies.

A total of 496 eyes of 332 patients with clinically diffuse nontractional diabetic macular edema (DME) underwent vitrectomy from August 1990 to June 2000 by the same surgeon (Nobuchika Ogino). There was no objective data using optical coherence tomography images.

All participants had undergone comprehensive ophthalmologic examinations, including measurements of the refractive error, best-corrected visual acuity (BCVA) measured with a standard Japanese chart in decimal units, axial length measurements, slit-lamp examinations, measurement of the intraocular pressure with a Goldmann applanation tonometer, and dilated indirect slit-lamp biomicroscopy with or without a contact lens.

The inclusion criteria included the presence of diffuse macular edema with an attached posterior hyaloid detected by slit-lamp biomicroscopy with a contact lens and no or mild cataract. Diffuse macular edema was detected as a central thickening of the retina and by the presence of diffuse fluorescein leakage involving most of the macular area in the fluorescein angiographic (FA) images. When a cyst larger than one-third the diameter of the optic disc was present, we diagnosed the eye as having cystoid macular edema.

Patients were included regardless of the metabolic control of diabetes, diastolic blood pressure, or visual acuity. The exclusion criteria were eyes with a thickened and taut vitreous membrane, a posterior vitreous detachment diagnosed by the presence of a Weiss ring, the presence of a vitreous hemorrhage, previous vitreous surgery, fibrovascular proliferation, epiretinal membrane, evidence of the vitreomacular traction syndrome, and ophthalmic disorders associated with macular edema such as uveitis and retinal vein occlusion, optic atrophy, or advanced glaucoma. Patients with chronic renal failure maintained by renal dialysis were also excluded.

Patients were examined preoperatively and postoperatively on day one, week one, month one, and months two, three, and six. Thereafter, the eyes were examined every 3 to 6 months.

All eyes were classified into four groups based on the length of the follow-up period (F): F <1 year (n = 10), 1 year ≦ F <5 years group (n = 128), 5 years ≦ F<10 years group (n = 201), and 10 years ≦F group (n = 157).

All surgeries were performed by one experienced surgeon (NO). A standard 20-gauge three-port pars plana vitrectomy was performed. Forty-one eyes with no previous panretinal photocoagulation (PRP) had intraoperative PRP. Ten eyes with insufficient PRP had additional intraoperative PRP. After removal of the anterior and central vitreous, a posterior vitreous detachment was induced by suction with a backflush needle. The posterior hyaloid membrane was removed by the backflush needle under passive aspiration. Phacovitrectomy with intraocular lens implantation was performed on phakic eyes. The internal limiting membrane (ILM) was peeled using ILM forceps in 181 (36.5%) of 496 eyes. The indications for initial surgery for large cysts and subretinal washout were based on the surgeon's decision. Both procedures were performed by balanced salt solution injection and removal using forceps through a temporal retinal incision. Recently, these procedures have been improved to be feasible for microincision vitreous surgery [31,35,36,38]. Triamcinolone acetonide was not used during surgery in any patients. The initial surgical procedures of the eyes by the length of the follow-up period are shown in Table 1. The distribution of surgical procedures was similar in all groups.

**Table 1:** Initial surgical procedures Data are expressed as number (%) ILM: internal limiting membrane, ICG: indocyanine green

Variables	Total	F<1Y	1Y≦F<5Y	5Y≦F<10Y	10Y≦F
N	496	10	128	201	157
Phacovitrectomy	442 (89.1)	10 (100.0)	118 (92.2)	184 (91.5)	144 (91.7)
ILM peeling	181 (36.5)	3 (30.0)	42 (32.8)	83 (41.3)	53 (33.8)
ICG staining	40 (22.1)	0	9 (21.4)	15 (18.0)	16 (30.2)
Surgery for cyst	57 (11.5)	0	18 (14.1)	22 (10.9)	17 (10.8)
Subretinal surgery	62 (12.5)	1 (10.0)	15 (11.7)	27 (13.4)	19 (12.1)

The decimal BCVA was converted to the logarithm of the minimal angle of resolution (logMAR) units for statistical analyses. The decimal BCVA was also converted to the corresponding Early Treatment Diabetic Retinopathy Study (ETDRS) letter scores. Paired numerical data were analyzed using t-tests. An increase or decrease in the visual acuity was defined as a change greater than 0.2 logMAR units. Statistical analyses of the data were performed using StatView software version 5.0 (SAS Institute, Inc., Cary, North Carolina, USA). A p < 0.05 was accepted as statistically significant.

## Results

The characteristics of the eyes by the length of the follow-up period are shown in Table 2. All eyes in the F<1 year group dropped out due to death and showed poor BCVA, shorter duration of diabetes, longer duration of edema, and higher hemoglobin A1c. Interestingly, the characteristics of the other three groups were similar.

**Table 2:** Characteristics of the study eyes Data are expressed as number (%) or mean ± standard deviation. F: follow-up, PRP: panretinal photocoagulation

Variables	Total	F<1Y	1Y≦F<5Y	5Y≦F<10Y	10Y≦F
N	496	10	128	201	157
Female	221 (44.6)	5 (50.0)	43 (33.6)	96 (47.8)	77 (49.0)
Male	275 (55.4)	5 (50.0)	85 (66.4)	105 (52.2)	80 (51.0)
Age (yrs)	60.2±9.8	66.5±10.5	61.8±10.9	60.6±9.9	58.2±8.1
Visual acuity	-	-	-	-	-
LogMAR units	0.72±0.47	1.0±0.54	0.79±0.53	0.70±0.46	0.68±0.42
ETDRS letter score	45±22	35±24	47±24	51±21	52±20
Duration of diabetes (yrs)	12.4±7.5	7.8±5.2	12.9±7.5	12.8±8.4	11.6±6.3
Duration of edema (mo)	11.4±11.3	20.4±22.2	12.3±12.2	10.8±10.6	10.9±10.4
Hemoglobin A1c	7.9±2.0	9.9±2.7	8.2±2.0	7.6±1.8	7.9±1.9
Hematocrit	38.0±5.3	38.9±3.1	37.3±6.1	38.1±5.5	38.2±4.5
Previous photocoagulation	-	-	-	-	-
Panretinal	453 (91.3)	8 (80.0)	113 (88.3)	188 (93.5)	144 (91.7)
Time since PRP (mo)	11.6±15.8	6.9±5.4	9.4±14.7	12.8±14.2	12.1±18.6
Macular grid	18 (3.6)	0 (0.0)	7 (5.5)	7 (3.5)	4 (2.5)
Lens status	-	-	-	-	-
Phakic	456 (91.9)	10 (100.0)	118 (92.2)	184 (91.5)	144 (91.7)
Pseudophakic	39 (7.9)	0	9 (7.0)	17 (8.5)	13 (8.3)
Aphakic	1 (0.2)	0	1 (0.8)	0	0
Protein urea	192 (38.7)	4 (40.0)	69 (53.9)	71 (35.3)	48 (30.6)
Cystoid macular edema	129 (26.0)	3 (30.0)	24 (18.8)	54 (26.9)	48 (30.6)
Subfoveal hard exdates	149 (30.0)	3 (30.0)	42 (32.8)	62 (30.8)	42 (26.8)
Follow-up (mo)	90.9±60.1	5.5±1.0	25.7±11.7	82.5±14.3	160.3±46.8
Lost to death	93 (18.8)	10 (100.0)	47 (36.7)	31 (15.4)	5 (3.2)

**Figure 1:**
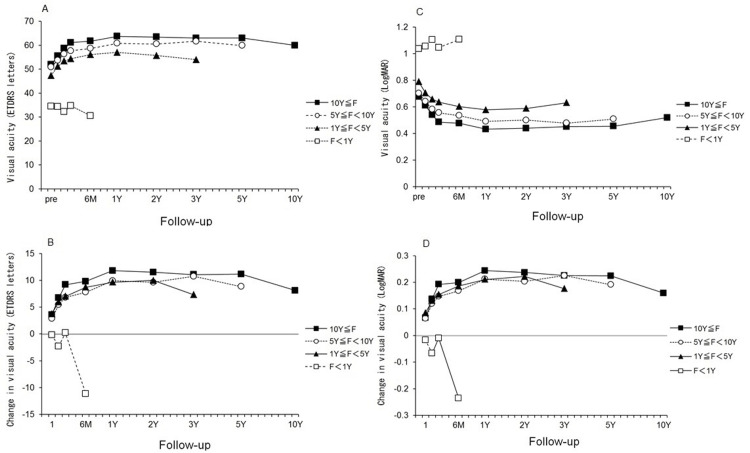
Time course of the mean visual acuity and changes from the preoperative visual acuity Time course of the mean visual acuity and changes from the preoperative visual acuity for the four follow-up period groups. The visual acuity is expressed as the Early Treatment Diabetic Retinopathy Study letter scores (Figures 1A, 1B) or the logarithm of the minimum angle of resolution (logMAR) units (Figures 1C, 1D).

The time course of the changes in the visual acuity from the preoperative visual acuity was separated by the duration of the follow-up period groups. The findings are expressed as ETDRS letter scores (Figures 1A, 1B) and in logMAR units (Figures 1C, 1D). There was a significant difference between preoperative BCVA and BCVA at all visits postoperatively in the BCVA for the three groups other than the F <1 year group (all, p <0.01) (Figure 1C). The time course of the changes in the BCVA for the three groups other than the F <1 year group were similar.

The postoperative complications and need for a reoperation are shown in Table 3. The characteristic complications in the 10-year ≦F group were atrophic creep, choroidal neovascularization, posterior ischemic optic neuropathy, renal dialysis, and cerebral infarction.

**Table 3:** Postoperative complications and reoperations Data are expressed as numbers

Variables	Total	F<1Y	1Y≦F<5Y	5Y≦F<10Y	10Y≦F
N	496	10	128	201	157
neovascular glaucoma	17	0	5	8	4
atrophic creep	19	0	0	7	12
secondary open angle glaucoma	26	0	3	16	7
branch retinal vein occlusion	1	0	1	0	0
choroidal neovascularization	1	0	0	0	1
central vein occlusion	2	0	1	0	1
epiretinal membrane	6	0	3	1	2
posterior ischemic optic neuropathy	1	0	0	0	1
subfoveal accumulation of hard exudate	26	0	3	15	8
dialysis	2	0	0	0	2
cerebral infarction	3	0	0	0	3
Reoperation	-	-	-	-	-
subretinal wash out	16	0	1	10	5
surgery for glaucoma	7	0	1	3	3
vitreous hemorrhage	10	0	2	4	4
retinal detachment	5	0	1	2	2
macular hole	2	0	0	1	1
suture of intraocular lens	1	0	0	0	1

Visual outcomes of the 10Y≦F group are shown in Table 4. The BCVA at 10 years after surgery improved in 80 eyes (51.0%), was unchanged in 47 eyes (30.0%), and decreased in 30 eyes (19.1%). The frequency of the decimal BCVA <0.1 was 24 eyes (15.3%), 0.1-0.4 was 58 eyes (36.9%), 0.5-0.9 was 38 eyes (24.2%), and ≧1.0 was 33 eyes (23.6%).

**Table 4:** Visual outcomes of the 10Y≦F group (n=157) Data are expressed as number (%) or mean ± standard deviation LogMAR: logarithm of the minimal angle of resolution, VA: visual acuity Improvement or worsening by 0.2 logMAR units

Variables	Pre	1 year	2 years	3 years	5 years	10 years
Visual acuity	-	-	-	-	-	-
ETDRS letter score	51.9±20.1	63.7±21.5	63.4±22.5	63.0±23.7	62.6±25.1	60.0±25.8
LogMAR units	0.68±0.42	0.43±0.45	0.44±0.47	0.45±0.51	0.45±0.55	0.52±0.56
VA change	-	-	-	-	-	-
ETDRS letter score	-	11.8±17.5	11.5±18.8	11.0±19.3	11.2±20.1	8.1±20.3
LogMAR units	-	0.24±0.36	0.24±0.39	0.23±0.41	0.22±0.44	0.16±0.44
improved	-	87 (55.4)	83 (52.9)	83 (52.9)	89 (56.7)	80 (51.0)
unchanged	-	54 (34.4)	55 (35.0)	54(34.4)	47 (30.0)	47 (30.0)
decreased	-	16 (10.2)	19 (12.1)	20 (12.7)	21 (13.4)	30 (19.1)
VA in decimal	-	-	-	-	-	-
<0.1	25 (15.9)	16 (10.2)	17 (10.8)	19 (12.1)	22 (14.0)	24 (15.3)
0.1-0.4	94 (59.9)	59 (37.6)	56 (35.7)	57 (36.3)	48 (30.6)	58 (36.9)
0.5-0.9	34 (21.7)	53 (33.8)	48 (30.6)	42 (26.8)	46 (29.3)	38 (24.2)
1.0≦	4 (2.5)	29 (18.5)	36 (22.9)	39 (24.8)	41 (26.1)	37 (23.6)

## Discussion

This study presents the findings after a 10-year follow-up period for cases of DME that had undergone vitrectomy by the same surgeon [20,25]. All eyes were classified into four groups based on the length of the follow-up period, and the postoperative outcomes were compared among the four groups. The F <1 year group was a unique group that included patients who had died within a year after the vitrectomy. The 10-year ≦F group appeared to represent the visual outcomes after vitrectomy for DME. The 10-year visual outcomes after vitrectomy appeared to be good and stable. In addition, we compared our results to the previous outcomes in Tables 5-7, 9-11. However, the use of the date in the tables has not been authorized by the original author.

Vitrectomy has been shown to improve the retinal oxygenation [45], increase the rate of intraocular cytokine turnover, remove the mechanical barriers to the outflow of fluids and metabolites, and increase the intraretinal penetration of intravitreally injected medications [4]. There have been reports that vitrectomy also improves the long-term stabilization of retinopathy [46] and the area of the foveal avascular zone [47] and improves the macular blood flow [48]. However, the exact role vitrectomy plays in the management of DME has not been definitively determined.

The relative costs and benefits should also be considered when developing and determining treatment strategies. Anti-VEGF treatment is more expensive than vitrectomy, but the professional fees are lower. The proportionate costs per category are 11% professional fees and 80% drug costs for ranibizumab treatments [7]. On the other hand, there is a 40% professional fee and a 60% technical fee for vitrectomy [7]. In addition, vitrectomy is widely used in regions of the world where economic resources are more limited and even in relatively affluent nations for underinsured patients [5].

Another advantage of vitrectomy is that treatment can be completed in a single procedure. Although some cases developed neovascular glaucoma postoperatively, such complications are now treatable with anti-VEGF injections. At a time when many ophthalmologists believe that multiple anti-VEGF injections are the first-line therapy for DME, we wish to emphasize that vitrectomy can provide long-term resolution of edema and visual improvement without requiring additional treatments.

It is difficult to compare anti-VEGF treatment and vitrectomy in diabetic patients because even if the visual acuities were the same, the general health conditions of the individuals can be different. Recently, Nawrocka et al. compared the results of vitrectomy performed on one eye to the results of the continuous use of anti-VEGF treatments of the fellow eye. They concluded that both techniques resulted in similar improvements in visual acuity and decreases in central retinal thickness after one year [43]. Although our study might have included cases of poor preoperative visual acuities, with poor DM control and advanced retinopathy, we have evaluated the visual outcomes of anti-VEGF treatments in four reports [12-14,49] by adapting the baseline visual acuity range and measurement year of the visual acuity.

Our visual outcomes and those reported by Glassman et al. [13] are shown in Tables 5, 6. The BCVA from the baseline to 2 years and to 5 years were 12.2 and 7.4 in their report, whereas they were 9.0 and 8.2 in this study. The degree of change of the BCVA between 2 and 5 years was -4.7, compared to -0.7 in this study. The rate of VA gains from baseline to 2 or 5 years tended to be higher in their report, whereas the rate of visual acuity loss from 2 and 5 years tended to be lower in this study. Even when the baseline VAs were divided into 20/50 to 20/320 and 20/32 to 20/40 groups, the trend was similar. These findings might indicate that the BCVA was stable after the vitrectomy.

**Table 5:** Visual acuity outcomes compared with the previous report The study included aflibercept (n=112), bevacizumab (n=96), and ranibizumab (n=104) Participants with visual acuity of 20/32 to 20/320 enrolled. VA: visual acuity. Data are expressed as number (%) or mean ± standard deviation. [13]

Variables	Glassman et al. [13]	Present study
Number of eyes	312	293
VA, letters	-	-
Baseline	65.7±11.0	54.7±14.2
Year 2	77.8±12.2	63.7±20.3
Year 5	72.8±16.4	63.0±22.7
5-Year VA Snellen group	-	-
20/25 or better (≥79 letters)	146 (47)	98 (33)
20/32 - 20/40 (69-78 letters)	83 (27)	59 (20)
20/50 - 20/160 (39-68 letters)	67 (21)	84 (29)
20/200 or worse (≤38 letters)	16 (5)	52 (18)
VA change from baseline to 5 years	-	-
≥15 letter improvement	94 (30)	123 (42)
10-14 letter improvement	54 (17)	37 (13)
5-9 letter improvement	50 (16)	35 (12)
±4 letter difference	66 (21)	39 (13)
5-9 letter worsening	17 (5)	12 (4)
10-14 letter worsening	8 (3)	7 (2)
≥15 letter worsening	23 (7)	40 (14)

**Table 6:** Visual acuity changes compared with previous report The study included aflibercept (n=112), bevacizumab (n=96), and ranibizumab (n=104) Participants with visual acuity 20/32 to 20/320 enrolled. Data are expressed mean ± standard deviation. VA: visual acuity [13]

Variables	Glassman et al. [13]	Present study
All Participants	-	-
Number of eyes	312	293
VA change, letters	-	-
Baseline to 2 years	12.2±12.3	9.0±18.1
Baseline to 5 years	7.4±14.9	8.2±20.8
Years 2 to 5	−4.7±12.1	−0.7±11.9
Participants with Baseline VA 20/50 to 20/320 (Letter Score 68 to 24)		
N	151	227
VA change, letters	-	-
Baseline to 2 years	16.8±14.0	10.4±19.2
Baseline to 5 years	11.9±16.6	10.0±21.4
Years 2 to 5	−4.8±14.1	−0.4±12.0
Participants with Baseline VA 20/32 to 20/40 (Letter Score 78 to 69)		
N	161	66
VA change, letters		
Baseline to 2 years	7.8±8.5	4.1±12.2
Baseline to 5 years	3.2±11.7	2.3±17.5
Years 2 to 5	−4.6±10.0	−1.8±11.4

Our visual outcomes and those reported by Elman et al. [12] are shown in Table 7.

**Table 7:** Comparison with visual outcomes at five years after intravitreal ranibizumab with prompt or deferred (≧24 weeks) focal/grid laser treatment Participants with visual acuity of 20/32 to 20/320 enrolled. Data are expressed as number (%). [12]

Variables	Prompt	Deferred	Present study
N	124	111	293
Visual acuity letter score at 5-yr visit	-	-	-
(approximate Snellen equivalent)	-	-	-
Median	76	77	69
≧79 (≧20/25)	53 (43)	44 (40)	98 (33)
78-69 (20/32-20/40)	32 (26)	39 (35)	59 (20)
68-59 (20/50-20/63)	18 (15)	14 (13)	42 (15)
58-49 (20/80-20/100)	9 (7)	9 (8)	25 (9)
48-39 (20/125-20/100)	5 (4)	3 (3)	17 (6)
≦38 (20/200)	7 (6)	2 (2)	52 (18)
Change in visual acuity (letter score)	8±13	10±13	8±21
Distribution of change	-	-	-
≧30 letters improvement	4 (3)	10 (9)	34 (12)
29-25 letters improvement	5 (4)	3 (3)	12 (4)
26-20 letters improvement	15(12)	11(10)	38 (13)
19-15 letters improvement	10 (8)	18 (16)	39 (13)
14-10 letters improvement	24 (19)	22 (20)	37 (13)
9-5 letters improvement	24 (19)	18 (16)	35 (12)
Same 4 letters	28 (23)	13 (12)	39 (13)
5-9 letters worse	3 (2)	7 (6)	12 (4)
10-14 letters worse	4 (3)	3 (3)	7 (2)
≧15 letters worse	7 (6)	6 (5)	40 (14)

The changes in the VA from the baseline to five years were eight for the prompt group, 10 for the deferred group, and eight for this study. The distribution of the BCVA at five years showed that the VA changes from the baseline to five years seemed to be similar. However, the data showed that in the vitrectomy group, the proportion of patients experiencing a decline of 15 letters or more is higher compared to the anti-VEGF group (Tables 5, 7). Characteristics of patients experiencing a decline of 15 letters or more are shown in Table 8. The causes of visual impairment were unclear in 22 eyes, atrophic creep in five eyes, neovascular glaucoma in three eyes, and foveal hard exudate in six eyes.

**Table 8:** Characteristics of eyes experiencing a decline of 15 letters or more at five years ILM: internal limiting membrane, CRVO: central retinal vein occlusion, NVG: neovascular glaucoma, RD: retinal detachment, HE: hard exudate

						Initial surgery							
No	Age	Sex	Duration	HbA1c	ILM	Cyst	Subretinal	Postoperative		Visual acuity in decimal			Follow-up
	(yrs)		of DM (yrs)	(%)	peeling	surgery	surgery	complications	Pre	Max	5 yrs	Final	(mo)
1	54	F	9	10.0	Yes	No	Yes	atrophic creap	0.09	0.40	0.01	0.01	125
2	54	F	9	9.1	No	No	No	None	0.10	0.06	0.03	0.03	125
3	73	M	10	7.0	No	No	No	None	0.10	0.03	0.03	0.03	65
4	49	F	10	7.0	No	No	No	None	0.10	0.10	0.01	0.08	129
5	64	M	20	8.6	No	No	Yes	None	0.10	0.10	0.04	0.04	96
6	54	F	9	10.0	Yes	No	Yes	CRVO	0.10	0.80	0.02	0.03	128
7	48	F	2	10.0	No	No	No	None	0.10	0.02	0.02	0.02	60
8	54	F	9	9.1	No	No	No	RD	0.10	0.05	0.02	0.02	126
9	57	M	7	8.0	No	Yes	No	None	0.10	0.15	0.01	0.01	144
10	62	M	10	5.9	Yes	No	Yes	optic neuropathy	0.10	0.70	0.04	0.02	210
11	64	F	10	7.0	Yes	No	Yes	NVG	0.10	0.15	0.03	0.04	81
12	53	F	4	8.6	No	No	Yes	atrophic creap	0.10	0.06	0.06	0.06	91
13	57	F	15	8.1	No	No	No	foveal HE	0.15	0.50	0.09	0.15	120
14	76	M	40	6.3	No	No	No	None	0.15	0.05	0.05	0.05	62
15	70	F	8	7.4	Yes	No	Yes	None	0.15	0.10	0.01	0.01	130
16	68	F	11	11.0	No	No	No	None	0.15	0.30	0.06	0.04	144
17	76	F	25	5.5	No	No	No	foveal HE	0.15	0.20	0.06	0.10	120
18	56	M	5	7.5	No	No	No	None	0.15	0.10	0.07	0.10	102
19	64	M	20	8.6	Yes	No	Yes	None	0.20	0.15	0.05	0.10	95
20	73	M	30	12.1	No	Yes	No	foveal HE	0.20	0.09	0.09	0.08	77
21	58	F	5	5.5	Yes	No	Yes	None	0.20	0.09	0.09	0.06	83
22	47	F	1	8.9	No	No	No	RD	0.30	0.10	0.10	0.10	73
23	62	F	13	9.2	No	No	No	None	0.30	0.30	0.10	0.08	159
24	51	M	14	7.6	No	No	No	None	0.30	0.10	0.10	0.10	61
25	57	M	12	6.7	Yes	No	Yes	atrophic creap	0.30	0.30	0.02	0.06	120
26	60	M	20	11.8	No	No	No	foveal HE	0.30	0.70	0.10	0.04	256
27	71	M	30	8.6	Yes	No	No	None	0.30	0.02	0.02	0.02	60
28	63	M	10	7.6	Yes	No	No	foveal HE	0.40	0.10	0.10	0.10	76
29	49	F	2	10.0	No	No	No	None	0.40	0.03	0.03	0.03	60
30	75	M	3	7.5	Yes	No	No	None	0.40	0.15	0.15	0.15	60
31	62	F	13	10.9	No	Yes	No	NVG	0.40	0.40	0	0	215
32	66	F	20	7.6	Yes	No	Yes	atrophic creap	0.50	0.10	0.10	0.04	95
33	62	F	20	9.0	Yes	Yes	No	None	0.50	0.10	0.10	0.10	100
34	48	M	1	7.3	Yes	Yes	No	None	0.50	0.90	0.15	0.10	222
35	57	F	12	9.8	No	No	No	None	0.50	0.60	0.20	0.10	151
36	62	M	20	7.2	No	No	No	None	0.60	0.20	0.20	0.20	61
37	64	F	20	9.5	No	No	No	None	0.70	0.20	0.20	0.10	86
38	66	F	11	7.8	No	No	No	foveal HE	0.70	0.09	0.09	0.08	78
39	74	M	24	11.8	Yes	No	No	NVG	0.70	1.00	0.01	0.01	84
40	60	F	35	9.1	Yes	No	No	atrophic creap	0.70	1.00	0.20	0.02	97

The use of anti-VEGF treatments for patients with good visual acuity is controversial [49-54]. Patients with better baseline VA are more vulnerable to vision reduction after anti-VEGF treatments [14]. Although there is no evidence, many clinicians initiate anti-VEGF treatments even when the visual acuity is only minimally affected or unaffected because of concern that the visual outcomes will be worse if treatment is deferred. Many reports have recommended only follow-up examinations for patients with good visual acuity [49-54].

Our visual outcomes and previous reports of patients with good visual acuity are shown in Tables 6, 9-11. Patients with visual acuity of 20/25 or better [49], 20/40 or better [14], 20/80 to 20/50 [14], and 20/32 to 20/40 [13] were studied in the three reports. The visual outcomes of this study seem to be similar to those of the earlier reports.

**Table 9:** Outcomes at two years for eyes with visual acuity of 20/25 or better Data are expressed as number (%) or mean ± standard deviation [49]

	Aflibercept	Present study
N	205	26
≧5 Letter decrease	33 (16)	3 (12)
Baseline visual acuity	-	-
Letter score	85.2±3.5	81.5±3.2
Snellen equivalent, mean	20/20	0.9
Change from baseline	0.9±6.4	0.4±6.6
≧5 Letter increase	55 (27)	8 (31)
≧10 Letter decrease	18 (9)	3 (12)
≧15 Letter decrease	5 (2)	1 (3.8)
Visual acuity at 2 years	-	-
Letter score	86.0±7.4	81.9±7.3
Snellen equivalent, mean	20/20	1
20/20 or better (≧84)	158 (77)	15 (58)
20/25 (83-79)	19 (9)	5 (19)
20/32 (78-74)	12 (6)	3 (12)
20/40 (73-69)	13(6)	2 (8)
20/50 to 20/80 (68-54)	1 (<1)	1 (4)
20/100 to 20/160 (53-39)	2 (<1)	0
20/200 or worse (38-0)	0	0

**Table 10:** Outcomes at three years with a baseline VA of 20/40 or better VA: visual acuity [14]

	Ciulla et al. [14]	Present study
Number of eyes	3282	98
Number of injections	14.6	0
Mean VA (letters)	76.3	75.8
Mean VA change (letters)	-3.5	2.6

**Table 11:** Outcomes at one, three, and five years with a baseline VA were between 54 and 63 letters (Snellen equivalent, 20/80 to 20/50) VA: visual acuity [14]

1 Year	Ciulla et al. [14]	Present study
Number of eyes	40832	62
Number of injections	6.2	0
Mean VA change (letters)	4.7	9.2
3 Years	-	-
Number of eyes	7728	50
Number of injections	15.4	0
Mean VA change (letters)	3.3	9
5 Years	-	-
Number of eyes	1192	48
Number of injections	26	0
Mean VA change (letters)	3.1	9.3

This study has several limitations, including its retrospective nature. We did not use optical coherence tomography to evaluate the morphological condition of the macula. Fluorescein angiographic images alone are not necessarily a good criterion to determine the feasibility of vitrectomy. FA alone cannot serve as a definitive criterion for determining the indication for vitrectomy. Nevertheless, in eyes with macular edema requiring treatment as confirmed by optical coherence tomography (OCT), many show fluorescein leakage on FA; thus, we believe that using FA as a reference was not entirely meaningless. Vitreoretinal traction may occur in the absence of a clinically thickened posterior hyaloid, and this study does not include evaluations of the macular thickness. Completion of the 10-year follow-up could be done in 157 of 496 eyes in this study, which makes a bias in the evaluation of the treatment. Advancements in surgical techniques since 2000, such as the introduction of minimum incision vitreous surgery, surgical adjuvants like brilliant blue dyes and triamcinolone, and other innovations. These advancements may improve surgical outcomes. Prospective blinded studies are needed to show the efficacy.

## Conclusions

Vitrectomy for clinically non-tractional DME has favorable outcomes over 10 years. Various treatments, including laser photocoagulation and steroid therapy, are currently expected to play a role in the treatment of DME. We believe that vitrectomy is one of the options for the treatment of non-tractional DME. Further studies are expected to compare the long-term effects of vitrectomy and anti-VEGF treatments.
